# Structural, Wetting and Magnetic Properties of Sputtered Fe_70_Pd_30_ Thin Film with Nanostructured Surface Induced by Dealloying Process

**DOI:** 10.3390/nano11020282

**Published:** 2021-01-22

**Authors:** Gabriele Barrera, Federica Celegato, Matteo Cialone, Marco Coïsson, Paola Rizzi, Paola Tiberto

**Affiliations:** 1 Advanced Materials Metrology and Life Sciences, INRiM, Strada delle Cacce 91, I-10135 Torino, Italy; f.celegato@inrim.it (F.C.); m.coisson@inrim.it (M.C.); p.tiberto@inrim.it (P.T.); 2 Department de Fisica, Universitat Autònoma de Barcelona, 08193 Cerdanyola del Vallès, Spain; matteo.cialone@uab.cat; 3 Chemistry Department and NIS, University of Turin, via Pietro Giuria, 7, I-10125 Torino, Italy; paola.rizzi@unito.it

**Keywords:** FePd thin film, dealloying process, wettability, magnetic properties

## Abstract

FePd alloys in the thin film form represent a multipurpose and versatile material with relevant chemical and physical properties studied in different research fields. Moreover, the ability to manipulate and fine-tune the film surface with nanometric scale precision represents a degree of freedom useful to adapt these thin film properties to the demands of different desired applications. In this manuscript, Fe_70_Pd_30_ (at. %) thin films are prepared with a thickness of 50 and 200 nm by means of the widely used co-sputtering deposition technique. Subsequently, selective removal of the iron element from the alloy and the consequent surface diffusion of the palladium was induced by a dealloying treatment under free corrosion conditions in hydrochloric acid. The size and shape of the grains of the as-deposited thin films determine the dissolution rate of the iron element with a direct consequence not only on the surface morphology and the stoichiometry of the alloy but also on the wetting and magnetic properties of the sample. X-ray diffraction, Scanning Electron Microscopy (SEM) images, contact angle and magnetic measurements have been performed to provide a thorough characterisation of the fundamental properties of these nanostructured bimetallic thin films.

## 1. Introduction

Bimetallic alloys with a magnetic transition element (Fe, Co, Ni) have attracted great attention in the field of scientific research for their peculiar and interesting properties [1,2,3,4]. The transition metal can be exploited directly for its intrinsic magnetic properties (e.g., magnetic separation) or to improve the alloy ability (e.g., enhancement of catalytic efficiency [5,6]) or as a sacrificial metal in the dealloying process to promote the formation of a porous nanomaterial with a large surface-to-volume ratio [7,8,9].

Among the thin-film bimetallic alloys of the iron group, FePt is intensively studied because it is recognized as a promising magnetic material for prospective applications in high-density magnetic storage devices and micro-electromechanical systems (MEMS) [10,11] because the ordered L1_0_ FePt possesses a very high magnetocrystalline uniaxial anisotropy. At the same time, FeCu is an attractive material to investigate for several devices and applications (e.g., as an electrode coating material for solar cell device [12]) both because it is magnetically soft properties with high magnetic moment per Fe atom and constituted by sustainable elements [4,13,14]; whereas co-sputtered FeAg granular alloy, composited of two non-mixing elements [15], have attracted much attention in research and device areas because of its giant magnetoresistance (GMR) properties [16,17].

Recently, FePd alloy have also attracted a growing interest in the scientific community because of their possibility to combine the properties of magnetic (Fe) and noble (Pd) metal elements in a multifunctional alloy [18,19,20,21,22]. The chemical composition of the FePd binary alloy system strongly influences its structure and magnetic properties allowing the use in a variety of applications [21,23,24,25,26]. These properties include magnetic shape memory effect for the Fe_70_Pd_30_ alloys [27,28,29,30,31], high perpendicular magnetic anisotropy for Fe_50_Pd_50_ [32,33], as well as hydrogen absorption characteristics for the palladium-rich FePd material [34,35], catalytic activities [36,37] and SERS devices [26].

Such bimetallic alloy materials are successfully produced in thin film form using different deposition technologies [38] to meet the current trends towards the miniaturization of materials and the reduction of the use of expensive and exhaustible element (e.g., Pd atoms). In thin films, distinct advantages over bulk materials [39] arise from their small thickness, large surface/volume ratio and unique structural properties that derive directly from the growth process. The unique properties of thin films have paved the way for new applications in the fields of integrated circuits, information technologies and sensors [38,40,41].

In addition, the ability to manipulate and fine-tune the film surface with nanometric scale precision has opened new opportunities in materials science [42]. Among the several physical and chemical methods used to nanostructure the film surface, the dealloying treatment is a common, fast and cost-effective process [43,44,45,46], fully suitable for the FePd alloy. This process is based on the selective removal of the less noble atoms from the alloy leading to structural, compositional and morphological transformations of the film surface with a consequent variation of its physical and chemical properties [9,47,48]. Generally, adequate control during the growth process and the subsequent dealloying process results in a fundamental role in determining the resulting properties of thin film.

This manuscript is focused on the study of the interplay between the wetting and magnetic properties of Fe_70_Pd_30_ thin film and the stoichiometry, morphology and spatial confinement of its surface which continuously evolve as a function of the dealloying treatment time.

These observed multiple degrees of freedom can be used to adapt the thin film properties to the needs of different desired applications.

## 2. Materials and Methods

FePd thin film was deposited at room temperature by dual-source radio-frequency RF sputtering (Cinquepascal, Milan, Italy) equipped with elemental iron (purity 99.99%) and elemental palladium (purity 99.98%) targets; using a confocal configuration, the iron target was driven at radio frequency whereas the palladium target with a continuous current. The deposition parameters were: base pressure of the sputtering at 4.0 × 10^−7^ mbar and, Ar gas pressure at 1.2 × 10^−2^ mbar. The deposition rate was experimentally evaluated (1.35 Å/s) and then later used to set the deposition time in order to obtain two samples with the desired thickness of 50 and 200 nm to obtain as-deposited samples with a marked difference in structural properties, see paragraph 3.1 below. The FePd thin films were deposited on a Si substrate covered by a SiO_2_ oxide (500 nm). The adhesion of FePd thin film on the SiO_2_ surface was ensured by a thin Ti(8 nm)/Au(80 nm) bilayer deposited between them.

Room-temperature grazing incidence X-ray diffraction (GIXRD) with Cu-K α radiation was used to study the crystal structure of the samples. The diffractometer was made by Panalytical in Malvern (UK). 

The as-deposited FePd samples were chemically treated for selected times in a 2 molar aqueous solution of hydrochloric acid under free corrosion condition in order to induce the dealloying process. The solution was prepared from reagents grade chemicals and de-ionised water.

Scanning Electron Microscopy (SEM) equipped by an energy dispersive x-ray spectrometer (EDS) was used to investigate the sample morphology and to evaluate the stoichiometry of the FePd alloy as a function of the dealloying treatment time. The SEM acceleration voltage was set to 20 kV. The stoichiometry results are affected by an uncertainty of 2%. The SEM was made by FEI company in Hillsboro (USA).

Investigation on the wettability properties of the surface of the FePd samples was performed at room temperature by means of contact angle measurements with a homemade-built setup. Each value results from the average of at least six measurements of a droplet of de-ionized water with a volume of 1 μL in different points of the sample surface.

Room temperature hysteresis loops were performed using an Alternating Gradient Field Magnetometer (AGFM) applying the magnetic field in the film plane in the interval −18 kOe ≤ H ≤ 18 kOe. The diamagnetic signal of the sample holder and film substrate was evaluated and adequately subtracted. The values of the coercive field were extrapolated from hysteresis loops; an error bar of 2 Oe was associated to take into account the uncertainty resulting from the measurement of the loop and from their subsequent extrapolation. The AGFM was made by Princeton Measurements Corporation in Princeton (USA). 

Atomic and magnetic force microscopy (AFM/MFM) to image the surface morphology and the magnetic domain configuration was performed in lift-mode using a commercial tip coated with a ferromagnetic CoCr alloy. The MFM images were acquired at the remanence state after the application of a saturating in-plane magnetic field. The AFM/MFM microscope was made by Bruker in Billerica (USA).

## 3. Results and Discussion

### 3.1. As-Deposited FePd Thin Film

The Fe:Pd ratio in the alloy was determined using EDS technique resulting in Fe_70_Pd_30_ for both samples. This evidence indicates that the stoichiometry of the alloy remains the same during the deposition independently of the sample thickness.

**Figure 1:**
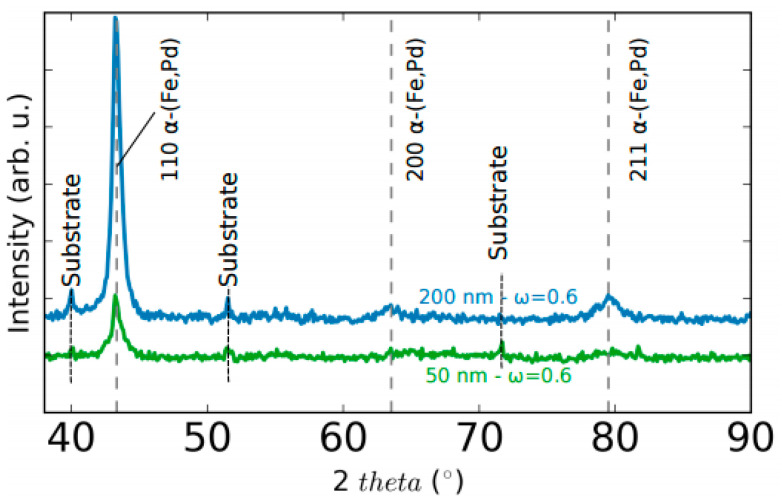
X-ray diffraction (XRD) patterns of the as-deposited S50 (green line) and S200 (blue line) samples.

The x-ray diffraction patterns of the as-deposited S50 and S200 samples are shown in Figure 1. Discerned the substrate peaks, both patterns indicate the formation of a metastable supersaturated solid solution of α-(Fe,Pd) [26,49]. The slight broadening of the reflection peaks indicates overlapping of diffraction peaks at slightly different angles induced by a non-uniformly distribution of the elements in the crystalline grains [26].

**Figure 2:**
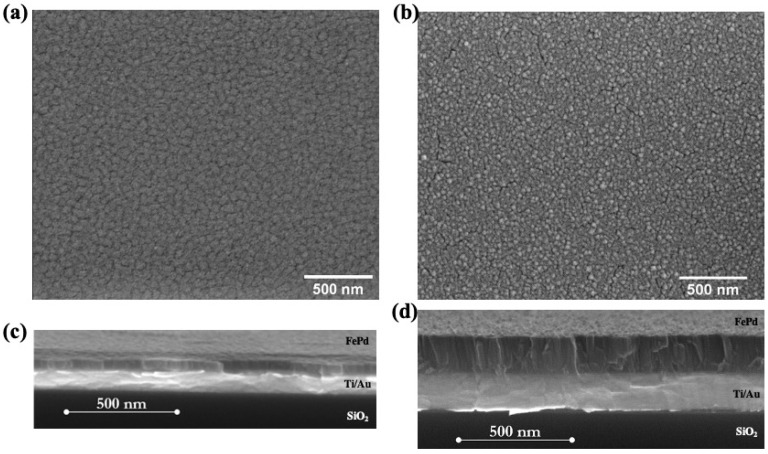
Top and cross section scanning electron microscopy (SEM) images of as-deposited S50 ((**a**,**c**) panels), S200 ((**b**,**d**) panels) samples.

The surface morphology of both as-deposited samples is shown in Figure 2a,b; the investigated area in the SEM images is the same to make easier the comparison. The S50 film surface appears conformal constituted by small, irregularly shaped and uniformly distributed grains with well-defined boundaries which are figured out as darker line around the grains [50,51]. The S50 sample cross-section observation (see Figure 2c) shows a homogeneous and dense growth of the thin film along the entire thickness. No crack is visible in the entire region under investigation.

By increasing the film thickness up to 200 nm, the surface morphology shows some narrow and short cracks induced by the accumulation of growing strain during the film deposition [52,53]. The S200 sample cross-section shows columnar crystals with well-visible boundaries oriented perpendicularly to the substrate [53]; see Figure 2d.

The crystalline grains’ dimension is estimated using the Scherrer formula, considering the full width at half maximum of the 110 peak of the α-(Fe, Pd) phase, obtaining for the S50 sample a <D>_50_ = (13 ± 2) nm and for the S200 sample a <D>_200_ = (12 ± 2) nm. Hence, in the range of thickness considered in this work, the dimensions of the crystalline grains are constant. Note that microstrains also contribute to peak broadening and thus the Scherrer’s formula underestimates crystal size.

**Figure 3:**
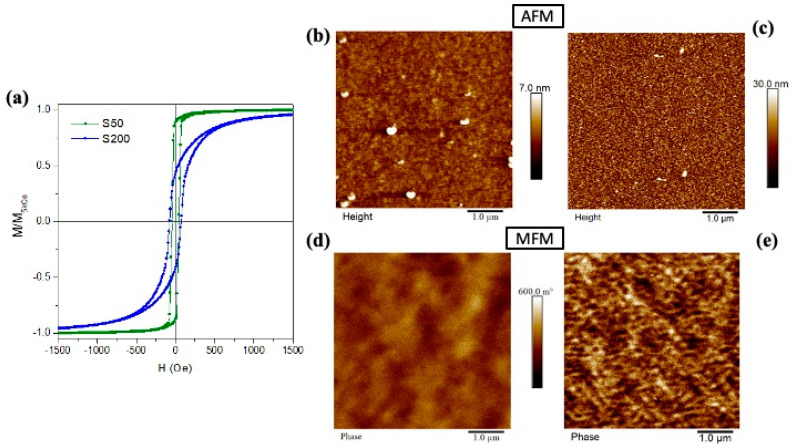
(**a**) Room temperature hysteresis loops of as-deposited samples; Atomic/Magnetic force microscopy (AFM/MFM) images of the as-deposited (**b**)/(**d**) S50 and (**c**)/(**e**) S200 samples. The MFM images are taken at parallel magnetic remanence.

Root-mean-square roughness (R_q_) values evaluated from the AFM images of Figure 3b,c are ~0.5 nm and ~4.3 nm for S50 and S200 samples, respectively. These values confirm the high flatness of the surface of the two as-deposited samples. The higher R_q_ value observed in the S200 sample denotes the more complex surface structure induced by columnar growth, as already observed in SEM images.

The wettability property of the as-deposited samples has been investigated by the contact angle (CA) values between a drop of deionized water and the sample surface itself. The measured CA values are 94° ± 3° and 97° ± 1° for S50 and S200 samples, respectively, see Figure 6. These results indicate a weak hydrophobic behaviour for both as-deposited samples where the effect of the different surface morphologies appears almost negligible. This evidence is probably because the observed differences in surface characteristics between the samples, in particular their very low roughness (Rq), determine only a minimal influence on the shape of the water drop so, presumably, only a slight variation of the CA value is induced, which is included in the measurement uncertainty.

Based on the Fowkes-Girifalco-Good (FGG) theory, the surface free energy $\left(\gamma_{s}\right)$ for the as-deposited FePd thin films can be calculated by the expression $\gamma_{s}=\;\frac{1}{4}\gamma_{l}\left(cos\theta_{CA}+1\right)$ where $\gamma_{l}$ is the liquid surface tension [54]. By using the deionized water ($\gamma_{l}=\;$72.8 mJ/m^2^) as tested liquid, the surface free energy of the as-deposited S50 and S200 samples are 16.9 and 16.0 mJ/m^2^, respectively.

Room temperature hysteresis loops of the as-deposited S50 and S200 samples are shown in Figure 3a; the magnetic field is applied parallel to the film plane and the curves are normalized to the magnetization value at H = 5kOe.

In the S50 sample, the reversal process of the magnetization is characterized by a single irreversible jump that occurs in a narrow field range; the coercive field (H_c_) and the normalized magnetization remanence (M_r_/M_s_) are H_c_ = 44 Oe and M_r_/M_s_ = 0.89. The magnetic domain configuration at the remanence state taken by the MFM (Figure 3d) appears as a slight modulation of the detectable out-of-plane component of the magnetization; it covers several grains resulting in a magnetically soft behavior with a high M_r_/M_s_ value and fast approach to saturation.

By increasing the sample thickness, the magnetization jump appears less intense and the reversal process covers a wider magnetic field range inducing a reduction of the M_r_/M_s_ = 0.43 and an increase of H_c_ = 73 Oe. In the MFM image of the S200 sample (Figure 3e), a more complex magnetic domain structure with a more evident light-dark contrast points out an out-of-plane component of the magnetization that periodically changes its sign. Such magnetic domains appear with an irregular pattern and its size is smaller than the one observed in the S50 sample. As a matter of fact, the columnar grains (see Figure 2b,d) induce a local crystal anisotropy energy resulting in a non-negligible effect on the orientation of the magnetization at the remanent state with a consequent increase of the out-of-plane component. In particular, a mostly randomly orientation in space of the magnetization, characterized by a low M_r_/M_s_ value, is obtained by a balance between the local anisotropy energy within each columnar grain against the shape anisotropy energy effecting in the film plane. Moreover, increasing the applied field performing the hysteresis loop, the field energy must overcome the local crystal anisotropy inducing a reversible rotation processes to bring the sample magnetization to saturation.

This interpretation about the magnetic behavior of the as-deposited samples, which is obtained from the analysis of the in-plane hysteresis loops and the MFM image, is also confirmed by the measured out-of-plane hysteresis loops shown in Figure S1 of Supplementary Materials.

Furthermore, the observed difference in H_c_ values between the two samples can also be related to the difference in crystalline structure and grain shape [55]. The counterplay between the local magnetic anisotropy inside the grain and the exchange interactions between adjacent grains determines the characteristics of the structure of the magnetic domains in the thin film, see Figure 3d–e. The grain boundaries acting as pinning sites [56] hinder the movement of the domain wall during the magnetization inversion process by increasing coercivity. It follows that the S200 sample, characterized by columnar grains, see Figure 3b,d, is characterized by a larger coercive field with respect to the S50 sample which displays a denser structure with less marked grains boundaries, see Figure 3a,c.

The main characteristics of the hysteresis loops observed in the as-deposited S50 and S200 samples are in good agreement with those already published in the literature about similar FePd or Fe thin films. The soft magnetic behavior characterized by the single irreversible magnetization jump in the inversion process was already observed by Cialone et al. [26] and by Liu et al. [57] in FePd thin film with a thickness of 100 and 30 nm, respectively. Instead, the progressive increase of the magnetic field interval in which the reversal process takes places and the consequent emergence in the hysteresis loop of reversible mechanisms leading to the magnetic saturation as a function of film thickness were previously observed by Salaheldeen et al. in FePd thin film on a glass substrate with thickness in the range 20–80 nm [58] and by Prida et al. in Fe thin film with a thickness in the range 30–100 nm [59].

### 3.2. Dealloying Process FePd Thin Film

**Figure 4:**
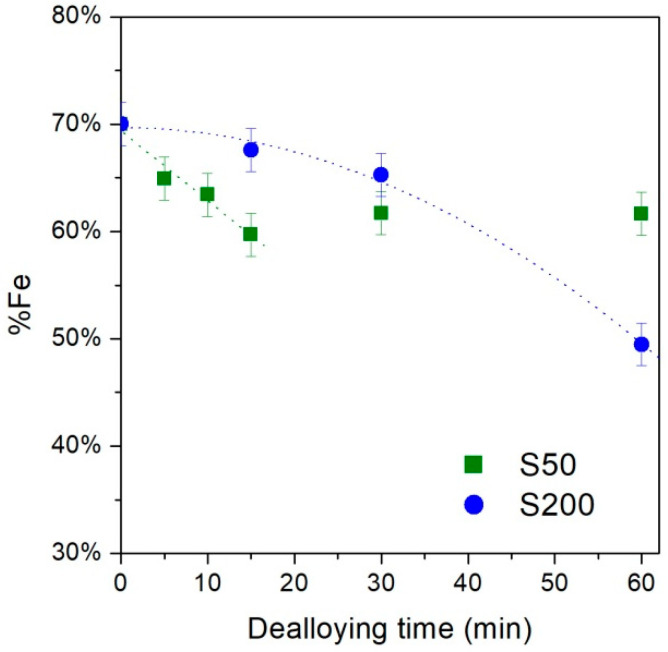
Evolution of Fe content as a function of the dealloying time in S50 and S200 samples.

The effect of the dealloying process on the stoichiometry of the FePd alloy as a function of the treatment time is observed to be different for the two studied samples (see Figure 4).

An almost linear dissolution rate of the Fe element is observed in the S50 sample up to 15 min (dashed green line), subsequently, the dissolution is stopped and the achieved alloy stoichiometry (Fe_60_Pd_40_) remains unaltered within the measurements uncertainty up to 60 min. This stop of the Fe dissolution effect is ascribed to the formation of a passivation layer rich in Pd on the film surface which hinders the further dealloying process on the FePd solid solution still presents. The formation of this passivation layer is favored by a high diffusion rate of the Pd atoms on film surface during the dealloying process [26,60].

Instead, the S200 sample shows a slower dissolution rate following a quadratic law (dashed blue line) and covers the entire time interval up to 60 min in which the alloy composition is Fe_50_Pd_50_ proving a dealloying process more effective. However, for treatment time longer than 60 min, the dealloyed FePd thin film detaches from the substrate preventing any further characterization, as a consequence the formation of a complete Pd passivation layer is not observed.

**Figure 5:**
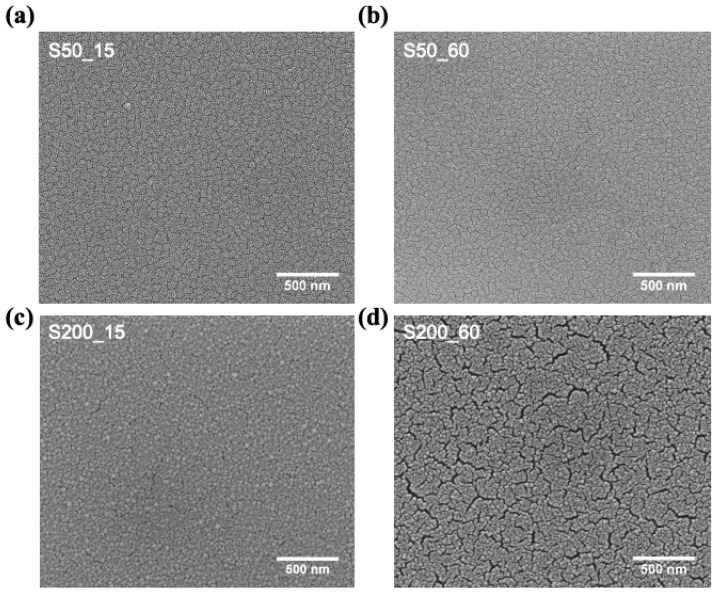
To-view SEM images: (**a**) S50_15, (**b**) S50_60, (**c**) S200_15 and (**d**) S200_60 sample.

The evolution of the sample morphology as a function of the dealloying time is shown in Figure 5a–d; the magnification of the reported SEM images is the same than the ones reported in Figure 2 for the as-deposited samples in order to make easier the comparison.

The grains of the dealloyed S50_15 sample appear with more evident boundaries (see Figure 5a) with respects to the as-deposited S50 sample. These features indicate that, in this sample, the dealloying process preferentially starts removing the Fe element from the grain boundaries whereas the core remains almost unaltered. Therefore, it can be deduced that the grain boundaries are less noble than the core of the grains, that is, they have a higher Fe content disposable for removal. The further increase in treatment time no longer affects the surface morphology (see Figure 5b for the sample S50_60) confirming the dealloying process is stopped after 15 min, as already observed by the EDS characterization (see Figure 4).

Instead, the very slow Fe dissolution rate at the beginning of the dealloying treatment observed by the EDS measurements of the S200 sample (Figure 4 blue line) indicates a homogenous stoichiometry and high electrochemical stability of its surface and, consequently, a slow evolution of its morphology is observed.

In fact, the morphology of the S200_15 sample (Figure 5c) appears almost unchanged with respect to the one of the S200 as-deposited sample (Figure 2b); the slight reduction of the Fe amount observed by EDS cannot revealed by the direct observation of sample surface. On the other hand, after 60 min of chemical treatment, the narrow and short cracks already visible in the as-deposited S200 (Figure 2b) and S200_15 (Figure 5c) samples appear wider and deeper (Figure 5d). Therefore, it is evident that the electrolyte penetrated the cracks and removed the Fe atoms by opening gradually wider voids and inducing a consequent increase of the Fe dissolution rate (Figure 4). Eventually, the fast iron removal will allow the electrolyte to penetrate the entire thickness of the film until it reaches the interface with the underlying gold. Here the corrosion process will determine the detachment of the film from the substrate for time longer than 60 min.

**Figure 6:**
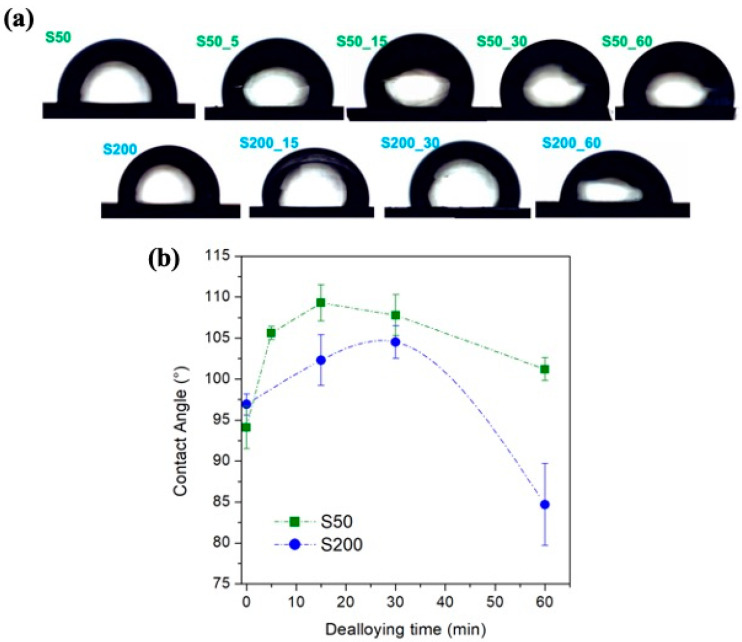
(**a**) Water drop on the sample surface; (**b**) contact angle evolution as a function of the dealloying time.

The surface properties such as stoichiometry, pattern and morphology play an important role to determine the wettability properties [54,61,62,63]. The drop placed on the sample surface and the measured contact angle (CA) values as a function of the dealloying time are reported in Figure 6 panel a and b, respectively; a hydrophobic behavior is observed in almost all samples.

It can be observed that the hydrophobic property increases with increasing the dealloying time up to 15 and 30 min for S50 and S200 sample, respectively. In the case of S50 samples, the maximum value of CA corresponds to the moment in which the dissolution of the Fe suspends, as observed in Figure 4 (green curve). For a longer treatment time, the measured CA values slowly decreases in S50 samples while strongly drops in S200 samples until to reach a hydrophilic behavior for the S200_60 sample.

Obviously, the behavior of the CA values is deeply related to the evolution of the stoichiometry and morphology of the sample surface [54,61,62,63] which continuously develop during the dealloying process. Although disentangle such contributes is difficult, in agreement with previous studies [54,62,64], the initial increase of the CA can be related to an increase in the surface patterning induced, in these studied samples, by the dealloying process. In particular, the surface patterning provides gaps where a large amount of water (Wenzel theory) or air (Cassie-Baxter theory) is trapped by changing the propensity of the drop to spread on the sample surface [62].

Following the FGG theory already used for the as-deposited samples [54], the surface free energy ($\gamma_{s}$) of dealloyed samples can be easily calculated from the measured CA values. The $\gamma_{s}$ values are included in the range 12.2–14.7 mJ/m^2^ for the S50 samples and in the range 13.6–19.9 mJ/m^2^ for the S200 samples.

**Figure 7:**
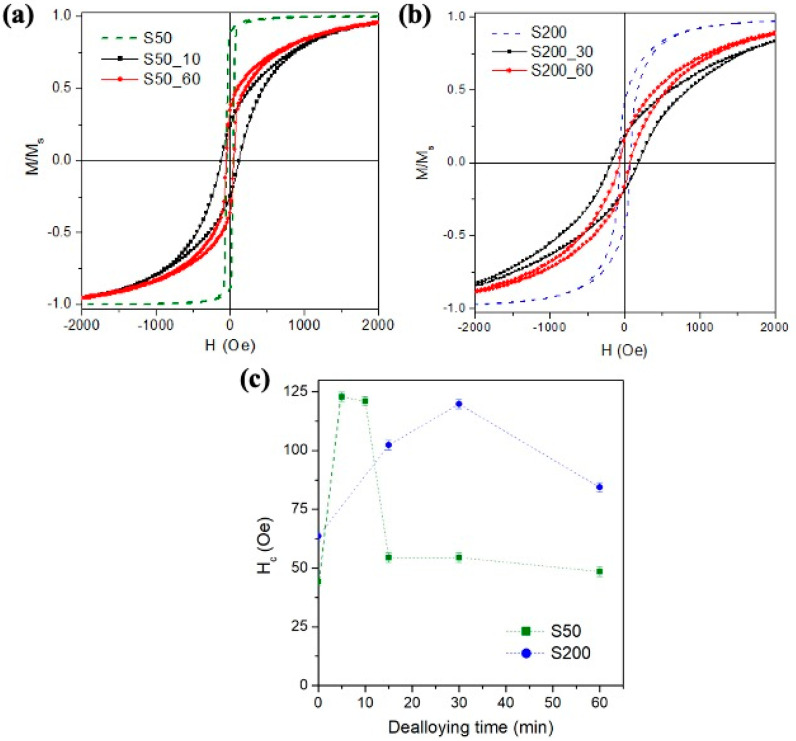
Room-temperature hysteresis loops for the in-plane direction of the field of (**a**) S50 samples and (**b**) S200 samples; (**c**) evolution of H_c_ as a function of the dealloying time.

The evolution of the room-temperature magnetic properties as a function of the dealloying time for the two studied samples is summarized in Figure 7. The chemical treatment implies some important changes in the shape of the hysteresis loops and in the coercive field value [26].

In general, the dealloying process broadens the range of the magnetic field in which complete magnetic saturation of the sample occurs. The irreversible processes are distributed over a larger portion of the hysteresis loop and the reversible rotation mechanisms occur at high field. Consequently, the value of the H_c_ increases in the treated sample whereas the one of M_r_/M_s_ decreases. These overall changes in magnetic behaviour induced by chemical treatment are in good agreement with those already published in the literature on similar systems: Sun et al. in Ni_70_Cu_30_ film [65], Robbennolt et al. in Fe_63_Cu_37_ [13] and in Fe_58_Cu_42_ thin films [66] show a development of reversible mechanisms in the reversal magnetization process, an increase of H_c_ values and a reduction of M_r_/M_s_ value induced by dealloying process performed in different conditions.

In particular, the single and sharp irreversible magnetization jump observed in the as-deposited S50 sample is completely disappeared after 10 min of dealloying treatment (Figure 7a); in fact, the loop branches of the S50_10 sample remain separated over a larger field interval indicating that the irreversible processes are now spread over a wider portion of the hysteresis loop. After the merging of loop branches, the complete magnetic saturation is reached at a higher applied magnetic field with a dominant role of the reversible rotation process. A further significant change compared to the S50 as-deposited sample is the increase of the coercive field for a treatment time up to 10 min, as shown in Figure 7c, with the arising of a non-negligible component of magnetization in the perpendicular direction with respect to the film plane as results from out-of-plane hysteresis loop shown in the Figure S2 of the Supplemental Materials.

Increasing the dealloying time, the shape of the hysteresis loop of S50_60 sample recovers some features more similar than the ones of the as-deposited sample: around the zero applied field region, a single, narrow although less marked irreversible jump of magnetization is visible; the coercive field and the merging field of the loop branches decrease their values close to the ones of the continuous thin film, as shown in Figure 7a.

Such evolution of the magnetic properties of the S50 samples as a function of the treatment time, which ends with the partial recovery of some magnetic features belonging to the as-deposited sample, allows formulating a hypothesis about the dissolution mechanism of Fe element during the dealloying process in this sample. As previously discussed, the dealloying process in the S50 sample preferentially starts from the boundaries of the grains by increasing their separation distance and making patterned the sample surface. Consequently, the magnetic decoupling among grains increases and local magnetic anisotropies, which hinder the domain wall motion during the magnetization reversal process, are originated. Such anisotropies favour the increase of the coercive field, the spreading of the irreversible processes over wider field range, the magnetization rotation process at high field, as observed for the hysteresis loop of the S50_10 sample and, the arising of a non-negligible component of magnetization in the perpendicular direction with respect to the film plane as results from out-of-plane hysteresis loop shown in the Figure S2 of the Supplemental Materials.

For longer treatment time, the EDS characterization has revealed that the formation of a passivation layer rich in Pd on the film surface stops the dealloying process. At this treatment time, the sample can be magnetically modelled as a bi-layered thin film: the upper layer is the passivation layer under which an almost unaltered Fe_70_Pd_30_ layer is still present; obviously, the interface between the layers is not clear and sharp but should be considered as a progressive transition region. As the upper rich in Pd layer can be considered magnetically inactive [67], the measured magnetization reversal of the S50_60 sample takes place in the interface region and mainly in the lower continuous Fe_70_Pd_30_ layer. This may result in a recovery of some magnetic features belonging to the as-deposited FePd thin film especially around at zero field range.

In the S200_30 sample, the decrease in M_r_/M_s_ and the increase in H_c_ compared to the as-deposited sample (see Figure 7b) are due to the patterning of the sample surface induced by the Fe dissolution process which involves mainly the surface cracks. Such patterning process arises local magnetic anisotropies that compete to the local crystal ones associated with the columnar grains hindering the domain wall motion during the magnetization process and making more randomly oriented in space the magnetization configuration at the remanence state as confirmed by the appearance of an out-of-plane component of magnetization in the out-of-plane hysteresis loop shown in the Figure S2 of the Supplemental Materials.

Although the EDS characterization of the S200 samples does not show a clear stop of the dealloying process, the magnetic properties of the S200_60 sample partially recover some features of the as-deposited S200 sample such as the value of H_c_ and the merging field of the loop branches (see Figure 7b,c). Similarly, to the S50 samples, also in the case of the S200_60 sample, it can be inferred that a passivation layer rich in Pd and magnetically inactive is arranging on the sample surface and the main contribution to the magnetic reversal process is due to the lower FePd layer almost unaltered by the dealloying process.

The order-of-magnitude estimation of the thickness of the passivation layer can be inferred under some rough assumptions: a net interface between the unaltered Fe_70_Pd_30_ and the upper passivation layer, which is now considered ideally constituted by pure Pd atoms only.

Under these assumptions, the EDS data reveal the ratio between the number of Fe atoms located only in the lower Fe_70_Pd_30_ layer and the number of Pd atoms present in both layers. During the dealloying process, the number of Fe atoms reduces due to the dissolution mechanism whereas the number of Pd atoms never changes. Therefore, the progressive reduction of the Fe content as a function of the dealloying process, as observed in the EDS data in Figure 4, can be interpreted as the progressive reduction of the thickness of the Fe_70_Pd_30_ substrate in favour of the formation of the passivation layer Pd. In this framework, from the EDS results obtained after 1 h of dealloying treatment (see Figure 4), the thickness of Pd passivation layer can be roughly estimated: ~20 nm and ~100 nm for S50 and S200 samples, respectively.

Although this estimation procedure is severely limited by the assumptions described above, it nevertheless confirms that the dealloying process is more effective in the S200 sample in which the electrolyte penetrated the cracks among the columnar structure compared to the S50 sample in which the deep Fe dissolution is hindered by the denser and more compact film structure.

The affinity of both samples in the structural evolution during the dealloying process, which ends with the formation of a passivation layer rich in Pd, is also underlined by the analogous evolution of H_c_, see Figure 7c. The initial increase in H_c_ is ascribed to the patterning of the sample surface induced by the Fe dissolution process, while the subsequent decrease in H_c_ corresponds to a progressive recovery of the characteristics of the as-deposited sample induced by the formation of the passivation layer. However, the comparison of the two curves shows how the rate of the dealloying process is different in the two samples, confirming the evidence obtained from the EDS characterization, see Figure 4. Although the maximum H_c_ value reached by the two samples is comparable, it is reached at much higher rates in sample S50 confirming the faster dissolution of Fe atoms in this sample rather than in S200. Similarly, after the H_c_ maximum, the decrease in H_c_ values is much more rapid in the S50 sample, indicating a faster formation of the passivation layer. These differences in the H_c_ curves can be mainly attributed to the initial difference in the structural properties of the as-deposited samples.

## 4. Conclusions

Continuous Fe_70_Pd_30_ thin films with thickness of 50 and 200 nm are deposited by a confocal sputtering allowing to easily control the film thickness by simply picking the desired deposition time. The as-deposited films display the same metastable supersaturated solid solution of α-(Fe,Pd) with a non-uniformly distribution of the Pd element in the crystalline grains. The film thickness does not affect the grains size but only their shape. The S50 sample appears homogeneous in thickness with a surface constituted by uniformly distributed grains with an average size of about ~13 ± 2 nm. Instead, the S200 sample displays columnar crystals with an average size of about ~12 ± 2 nm; moreover, the accumulation of growing strain during the film deposition induces the formation of narrow and short cracks. Such morphological differences between the as-deposited samples do not affect the wettability properties which results in weak hydrophobic behaviour for both samples with similar surface free energy values. The magnetic properties of the continuous thin films are in good agreement with their observed morphology: the columnar grains of the S200 sample induce a more randomly orientation in space of the magnetization than the more uniform structure of the S50 sample resulting in a lower M_r_/M_s_ value, a smaller magnetic domain size and a wider magnetic field range in which the magnetization reversal occurs.

As-deposited Fe_70_Pd_30_ samples show different responses to the dealloying treatment under free corrosion condition in hydrochloric acid (2 M aqueous solution of HCl) as a function of film thickness indicating a different distribution of the elements in the crystalline grains. The combination between the removal of the Fe and the diffusion of the Pd atoms has allowed modifying the surface features, which turns out to play a fundamental role in determining the resulting properties of the samples such as the wetting and magnetic ones. The Fe element was preferentially removed from the grain boundaries in the S50 sample while the electrolyte preferentially acts inside the cracks opening gradually wider and deeper voids in the S200 sample. In the S50 sample, the high diffusion rate of the Pd atoms on the film surface favors the formation of a passivation layer rich in Pd which stops the dealloying process.

The wettability properties of the samples are deeply related to the evolution of the stoichiometry and morphology of the sample surface during the dealloying process. The surface patterning induced by the dealloying treatment provides gaps which change the propensity of the drop to spread on the sample surface with the surface free energy values included in the range 12.2–14.7 mJ/m^2^ for the S50 samples and in the range 13.6–19.9 mJ/m^2^ for the S200 samples. The dealloying treatment also implies some important changes in magnetic properties. The irreversible processes of magnetization are spread over a larger portion of the hysteresis loop, the complete magnetic saturation is reached at a higher applied magnetic field and the coercive field increases. However, a recovery of some magnetic features more similar to those of the as-deposited samples is observed in the samples chemically treated for a longer time. In these samples, the main contribution to the magnetic reversal process stems from a portion of FePd thin-film almost unaltered respect to the as-deposited one because it is covered by the passivation layer rich in Pd and magnetically inactive arranged on the sample surface during the dealloying process. The development of the Pd-rich passivation layer can also be exploited as a protective layer allowing the underlying FePd alloy to maintain its magnetic properties stable over time even if the sample will be used in an acid environment.

## Data Availability

The data presented in this study are available on request from the corresponding author.

## Supplementary Materials

The following are available online at https://www.mdpi.com/2079-4991/11/2/282/s1, Figure S1: Out-of-plane room temperature hysteresis loops of the as-deposited S50 and S200 samples, Figure S2: Out-of-plane room temperature hysteresis loops of the S50_10 and S200_30 samples.
