# Supplementary material for: Structural, Wetting and Magnetic Properties of Sputtered Fe70Pd30 Thin Film with Nanostructured Surface Induced by Dealloying Process
Source: Nanomaterials (Basel). 2021 Jan 22;11(2):282. doi: 10.3390/nano11020282 (PMC7911447; doi:10.3390/nano11020282)
Supplement: Supplementary file 1 [file nanomaterials-11-00282-s001.pdf]

Supplementary Materials

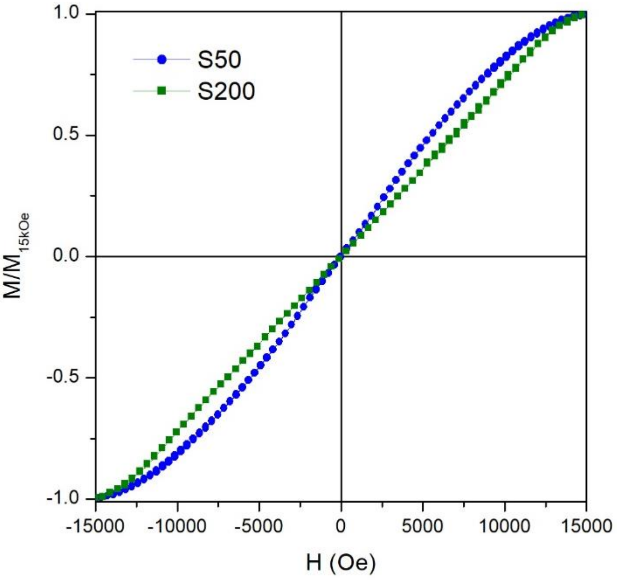

Figure S1: Out-of-plane room temperature hysteresis loops of the as-deposited S50 and S200 samples.

Out-of-plane room temperature hysteresis loops of the as-deposited S50 and S200 samples are shown in Figure S1; the magnetic field is applied in the perpendicular direction with respect to the film plane. Both curves are normalized to the magnetization value at H = 15kOe, which is the maximum applicable magnetic field by our Alternating Gradient Field Magnetometer.
The shape of both hysteresis loops figures out that the perpendicular direction is the hard axis, along which a large applied magnetic field (higher than 15 kOe) is needed to reach the saturation magnetization.
The magnetic moments align to the magnetic field direction mainly by means of a rotation process as indicated in the hysteresis loops by the linear approaching to saturation without coercivity.

The higher slope of the linear section of the hysteresis loop in the S200 with the appearance of a barely noticeable sigmoidal shape indicates a slightly "easier" achievement of saturation magnetization in this sample than in the S50 sample. This observation, even more visible if the normalization of the curves were carried out at real saturation magnetization and not only at H = 15 kOe, indicates that the easy axis of magnetization is completely in the film plan in the S50 sample whereas it is slightly tilted in the S200 sample with a minor but non-negligible out-of-plane component. This evidence is in good agreement with the in-plane hysteresis loops and MFM characterization reported in the main manuscript; see Figure 2 and related comments.

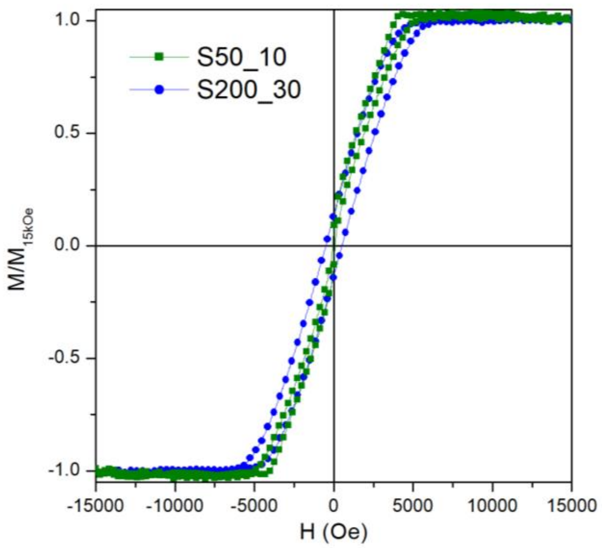

Figure S2: Out-of-plane room temperature hysteresis loops of the S50_10 and S200_30 samples.

Out-of-plane room temperature hysteresis loops of the dealloyed S50_10 and S200_30 samples are shown in Figure S2; the magnetic field is applied in the perpendicular direction respect to the film plane. Both curves are normalized to the magnetization value at H = 15kOe. The magnetic field values, at which the complete saturation occurs, are significantly lower respect to ones observed in the corresponding as-deposited S50 and S200 samples, see Figure S1. This evidence indicates that the increase of magnetic decoupling among grains and the consequent arising of local magnetic anisotropies (see main text for details) with an out-of-plane component of magnetization which describes well-defined hysteresis loops. The coercive field values are ≈ 80 and ≈ 510 Oe in S50_10 and S200_30, respectively.
